# Seed Germination of Three Milk Thistle (*Silybum marianum* (L.) Gaertn.) Populations of Greek Origin: Temperature, Duration, and Storage Conditions Effects

**DOI:** 10.3390/plants12051025

**Published:** 2023-02-24

**Authors:** Vasiliki Liava, Georgia Ntatsi, Anestis Karkanis

**Affiliations:** 1 Laboratory of Weed Science, Department of Agriculture Crop Production and Rural Environment, University of Thessaly, Fytokou St., 38446 Volos, Greece; 2 Department of Crop Production, Agricultural University of Athens, 11855 Athens, Greece

**Keywords:** genotypes, integrated weed management, milk thistle, seedbank, weed

## Abstract

Milk thistle besides being a highly competitive weed is cultivated as a medicinal plant, and the seeds of which have been clinically utilized in several disorders caused in liver. The present study aims to evaluate the effect of duration and storage conditions, population, and temperature on seed germination. The experiment was conducted in Petri dishes with three replications and three factors: (a) wild populations of milk thistle (Palaionterveno, Mesopotamia, and Spata) originating from Greece, (b) duration and storage conditions (5 months at room temperature, 17 months at room temperature, and 29 months in the freezer at −18 °C), and (c) temperature (5 °C, 10 °C, 15 °C, 20 °C, 25 °C, and 30 °C). All three factors significantly affected germination percentage (GP), mean germination time (MGT), germination index (GI), radicle length (RL), and hypocotyl length (HL) and significant interactions among the treatments were noted. In specific, no seed germination was recorded at 5 °C, while the populations showed higher GP and GI at 20 °C and 25 °C after 5 months of storage. Prolonged storage negatively affected seed germination although, cold storage mitigated this effect. Moreover, higher temperatures reduced MGT and increased RL and HL with the populations reacting differently in storage and temperature regimes. The results of this study should be taken into consideration when proposing the appropriate sowing date and storage conditions of the seeds used as propagation material for crop establishment. Moreover, the effects of low temperatures such as 5 °C or 10 °C on seed germination as well as the high decline rate in germination percentage over time could be utilized in the design of integrated weed management systems thereby indicating the importance of the sowing time and the suitable crop rotation system to weed control.

## 1. Introduction

*Silybum marianum* (L.) Gaertn., known as milk thistle due to its milky sap [1], belongs to the Asteraceae family and is considered both a common weed in many crops and a medicinal plant [2,3,4]. As a weed, milk thistle is characterized very competitive as it develops rosette with large leaves and stems of great height, while it spreads rapidly [2,3,5]. This weed species can cause high yield loss in wheat crops ascribed to competition for nutrients throughout the growing period [3,6]. However, milk thistle is also an important crop as its seeds contain silymarin, a bioactive complex of flavonolignans, widely known for its pharmaceutical potentials [4,7,8]. Hepatoprotective, anticancer, antioxidant, antifibrotic, and anti-inflammatory properties of silymarin have been reported [9,10,11].

Seed germination is essential not only for the survival and dispersal of plant species but also for crop establishment and depends on the dormancy of the seed [12,13] which is regulated by internal (e.g., abscisic acid) and environmental factors [14,15]. Milk thistle seeds are characterized by a non-deep physiological dormancy, while the type of this process is determined by the environmental conditions that prevail during seed development in the mother plant [16]. In many plant species, milk thistle included, light and temperature are important factors that affect both seed dormancy and germination [15,17,18]. In milk thistle, light could increase seed germination by 15 to 39.7% [5], while the base, optimum, and maximum temperatures for seed germination of this weed species are 5.2 °C, 24 °C, and 34.3 °C, respectively [19]. Moreover, Montemurro et al. [5] observed that the germination percentage of milk thistle seeds was higher (70–86.6%) at 25 °C or 30 °C constant temperature, or when schemes of 25 °C for 16 h and 15 °C for 8 h were applied.

Weed establishment is determined by seed dormancy level and germination [20,21]. Thus, research on seed persistence and germination is key for understanding the germination ecology of weeds. The knowledge in this area is important as it can contribute to improving the effectiveness of control methods [22] as well as the development of integrated weed management programs [23].

Moreover, seed quality impacts crop establishment and yield [24]. Thus, seed storage under suitable conditions is a very important practice in order to extend its viability. Both storage duration and conditions can affect seed quality, viability, and germination [25,26]. For instance, prolonged storage at room conditions can decrease the seeds germination in various crops such as einkorn (*Triticum monococcum* L. subsp. *monococcum*) [25] and soybean (*Glycine max* L.) [26], while seed storage under cooling conditions can retain high germination rate [26]. Regarding the germination percentage, differences between genotypes are also reported in various plant species [16,26]. In a recent study, Islam et al. [27] reported significant differences between 14 industrial hemp (*Cannabis sativa* L.) varieties with the greatest germination percentage (70%) recorded in the variety Han FNQ. Variation in germination percentage is also observed between three milk populations (P1, P2, and P3) [16]. Moreover, Koskosidis et al. [26] observed significant differences between soybean varieties in the germination percentage of seeds stored for different duration indicating different tolerance of the varieties to storage. It is also important to point out that the environmental conditions prevailing during the growth of mother plants can affect the seeds germination of various plant species such as common vetch (*Vicia sativa* L.) and black locust (*Robinia pseudoacacia* L.) [28,29]. Roman et al. [29] observed significant differences in the seed germination percentage between black locust genotypes originating from eight Romanian regions indicating the impact of maternal environment on this parameter. Moreover, seed dormancy level is also affected by both the genotypes and environmental conditions during the seed development in mother plants [30,31]. Recently, Fernández et al. [31] reported that the lowest seed dormancy level of perennial ryegrass (*Lolium perenne* L.) was induced by high temperatures (a great number of days with maximum temperatures > 29 °C) prevailing during the seed filling.

Based on the above-mentioned fact, the assessment of seed germination of milk thistle is an important issue as this species can be studied both as a weed and a medicinal crop. Thus, the present study aimed to evaluate the effect of temperature, duration, and storage conditions on seed germination of three milk thistle populations of Greek origin.

## 2. Results

Duration and storage conditions, populations, and temperature significantly affected germination percentage, mean germination time, germination index, radicle length, and hypocotyl length. Significant interactions among the three factors and all the possible factor combinations were observed only in germination percentage and germination index (Table 1). Moreover, interactions between all the combinations of the two factors were also recorded in mean germination time. For hypocotyl length, significant interactions between population and temperature as well as storage and temperature were noted, while for radicle length the only significant interaction was between storage and temperature.

### 2.1. Germination Percentage

**Figure 1:**
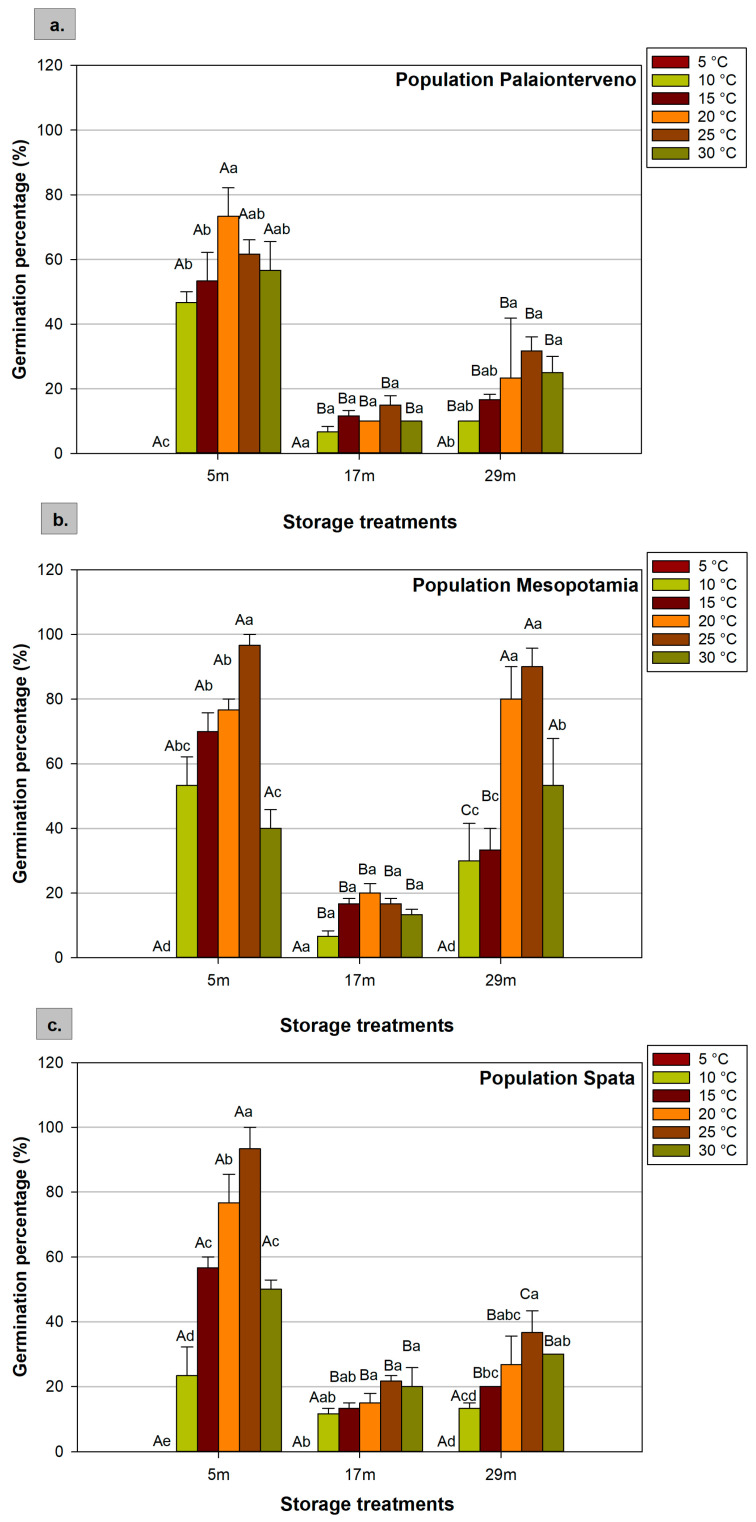
Effect of population ((**a**) Palaionterveno, (**b**) Mesopotamia, and (**c**) Spata), storage (duration and conditions: 5, 17, and 29 refer to the months (m) of storage and the conditions that prevailed during storage), and temperature (5 °C, 10 °C, 15 °C, 20 °C, 25 °C, and 30 °C) on milk thistle seeds’ germination percentage. Capital letters show significant differences between storage treatments at the same temperature, while small letters show significant differences between temperatures at the same storage treatment. The bars represent the standard error of the means.

The three experimental factors influenced the germination percentage of milk thistle seeds, while an interaction effect between the three factors was found. When assessing the temperature as an individual factor, the maximum germination of seeds stored for 5 months at room temperature of all three populations was recorded at 20 °C and 25 °C without any significant difference between the two temperatures (Table 2). Moreover, no seed germination at 5 °C was recorded, regardless of the population and the storage duration applied. Regarding the interaction effect between the factors, the highest percentage (96.7%) was recorded at 25 °C in the Mesopotamia population for the seeds that were stored for 5 months at room temperature (Figure 1b). Similarly, in the population Spata, 5-months storage and 25 °C were the most favorable conditions for seed germination (Figure 1c). However, in the population Palaionterveno, the highest germination (73.3%) was observed at 20 °C after 5 months of storage. For seeds that were stored for 5 months at 25 °C, the lower germination was recorded in the Palaionterveno (Figure 1a) population compared to the other two populations, although the reverse was the case at 30 °C for the same storage duration.

Moreover, at 20 °C, the germination percentage (mean values of three populations) of seeds stored for 5 months ranged from 73.3% to 76.7% (Table 3). However, the population Spata had significantly lower germination at 10 °C compared with the Palaionterveno and Mesopotamia populations. In general, prolonged storage significantly reduced germination percentage. For instance, for the seeds that were stored for 17 months, the germination percentage was 15%, even at the optimum temperature of 20 °C (Table 4). After 17 months of storage at room temperature, the seeds of all populations displayed lower germination as the percentages ranged from 6.7% to 21.7%. At these storage conditions, the population Palaionterveno had lower germination compared to the other populations (Figure 1). However, the germination percentage of seeds stored for 29 months at −18 °C was greater than in the 17 months of storage at room temperature. The effect of cold storage was more noticeable in the population Mesopotamia where the germination percentages were almost at the same level as for the seeds stored for 5 months (Figure 1b).

### 2.2. Mean Germination Time

**Figure 2:**
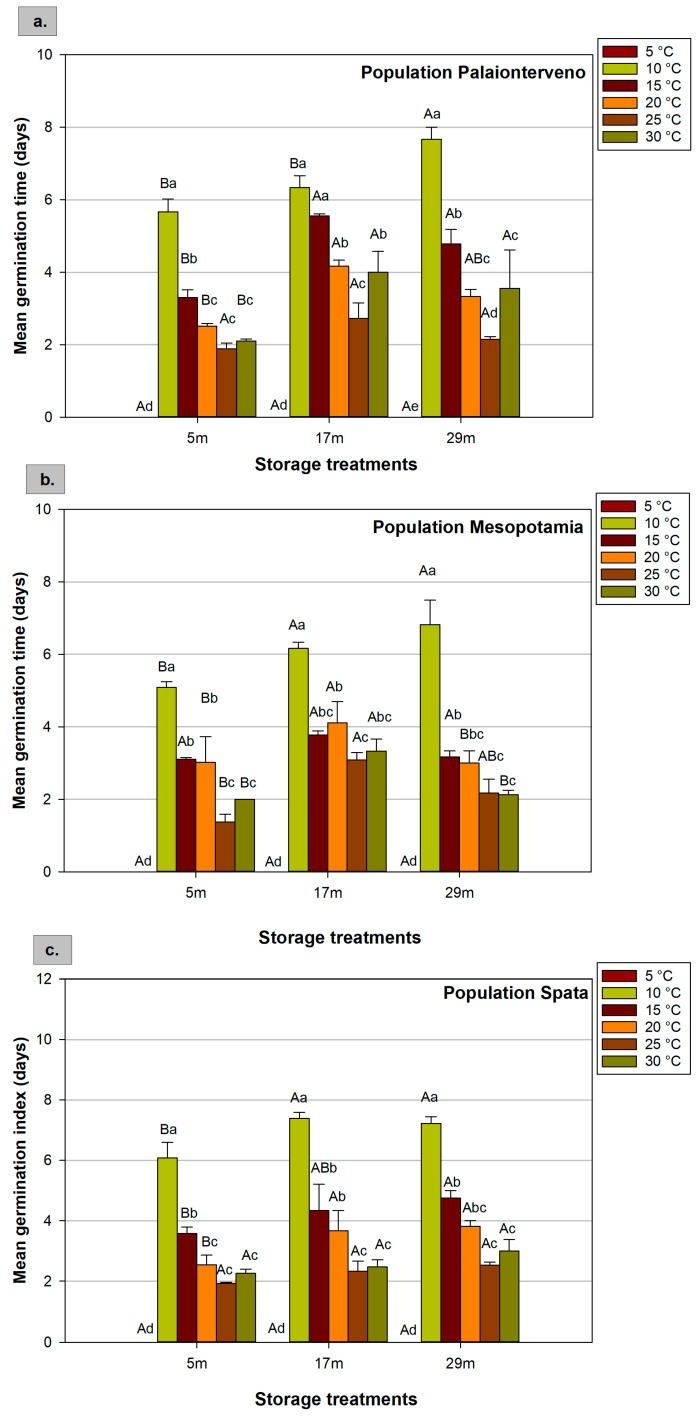
Effect of population ((**a**) Palaionterveno, (**b**) Mesopotamia, and (**c**) Spata), storage (duration and conditions: 5, 17, and 29 refer to the months (m) of storage and the conditions that prevailed during storage), and temperature (5 °C, 10 °C, 15 °C, 20 °C, 25 °C, and 30 °C) on milk thistle seeds’ mean germination time. Capital letters show significant differences between storage treatments at the same temperature, while small letters show significant differences between temperatures at the same storage treatment. The bars represent the standard error of the means.

The mean germination time of milk thistle ranged from 1.37 to 7.67 days and was significantly affected by the three experimental factors (Figure 2). In general, lower temperatures such as 10 °C and 15 °C led to significantly higher mean germination time. For instance, taking the mean values of the three populations for the seeds that were stored for 5 months at room temperature, the mean germination time was 5.61 and 1.73 days for 10 °C and 25 °C, respectively (Table 2). Moreover, after 29 months of cold storage, in the population Palaionterveno, the increase in incubation temperature from 10 °C to 25 °C reduced the mean germination time by 72% (Figure 2a). At 20 °C, there was no significant difference between the three populations after 5 months of storage at room temperature (Table 3). Similarly, the mean germination time of the three populations was not significantly different even at 25 °C, and this temperature is the optimum for milk thistle germination.

Moreover, taking into consideration the interaction effects, at 30 °C, the Palaionterveno population had a higher mean germination time and was affected by storage duration and conditions. In general, prolonged storage increased the mean germination time as there were significant differences between the same population at different storage conditions and duration. In particular, in the population Mesopotamia, at 25 °C, the mean germination times were 1.37 and 3.08 days for seeds that were stored at room temperature for 5 and 17 months, respectively (Figure 2b). However, when the germination was tested at the optimal temperature as the mean values of the three populations, the differences between the storage duration and conditions cannot be considered significant (Table 4).

### 2.3. Germination Index

**Figure 3:**
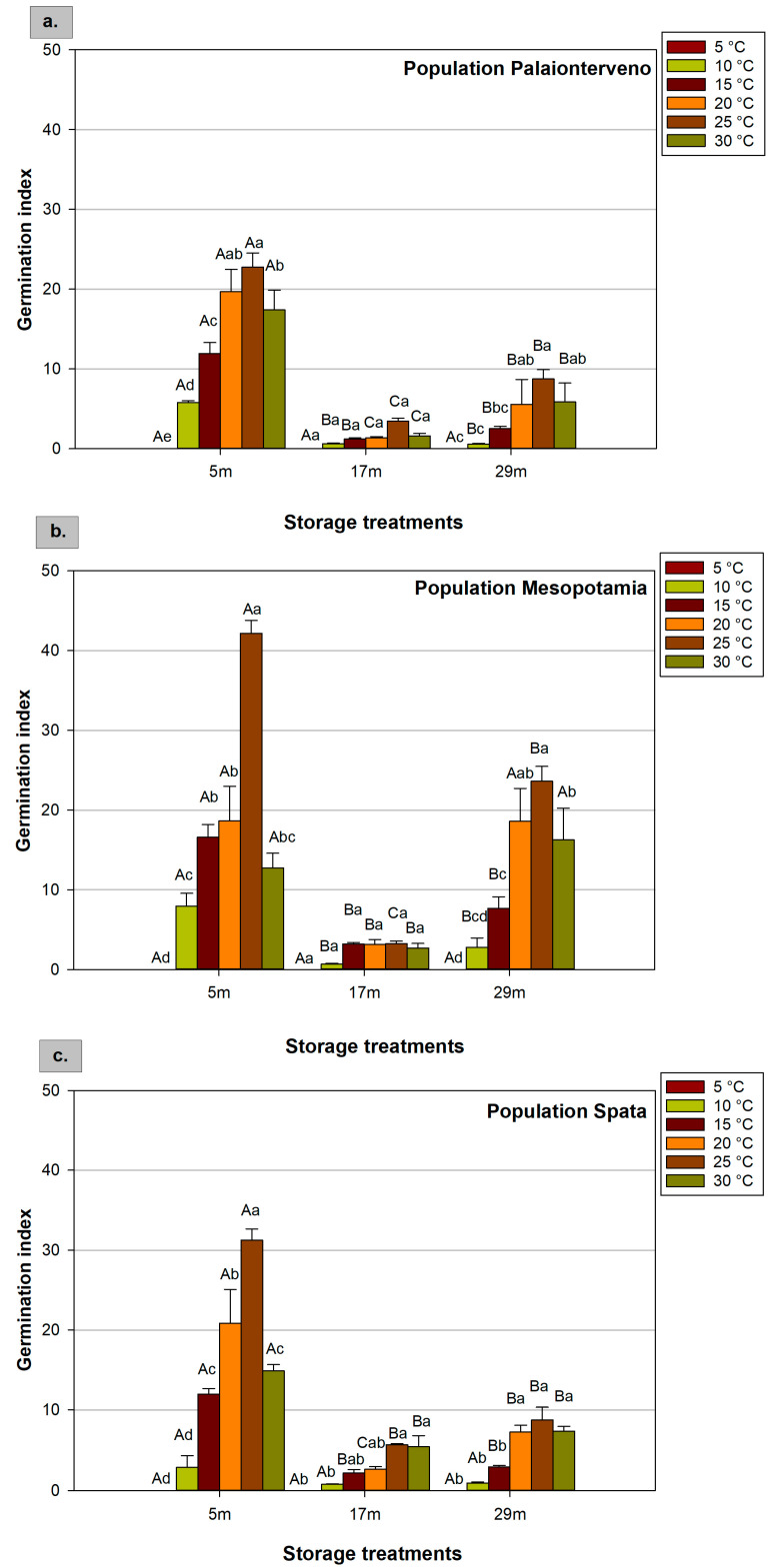
Effect of population ((**a**) Palaionterveno, (**b**) Mesopotamia, and (**c**) Spata), storage (duration and conditions: 5, 17, and 29 refer to the months (m) of storage and the conditions that prevailed during storage), and temperature (5 °C, 10 °C, 15 °C, 20 °C, 25 °C, and 30 °C) on milk thistle seeds’ germination index. Capital letters show significant differences between storage treatments at the same temperature, while small letters show significant differences between temperatures at the same storage treatment. The bars represent the standard error of the means.

The influence of population, storage, and temperature on the germination index was noticeable (Figure 3). At 20 °C, after 5 months of seed storage at room temperature the differences between the three populations were insignificant (Table 3). However, at 25 °C, the highest index (42.2) was recorded in the population Mesopotamia, when seeds were stored for 5 months and was significantly higher compared to the other temperature and storage treatments (Figure 3b). For this storage condition, in the population Mesopotamia, the effect of the temperature was stronger at 25 °C compared with the other populations, although at 20 °C and 30 °C, the populations Palaionterveno and Spata showed higher germination index, indicating the importance of the interaction effect (Figure 3a,c). For seeds that were stored for 5 months, the highest germination index (mean values of three populations) was observed at 25 °C and was significantly different from the other temperatures (Table 2). Considering storage duration and conditions, 5 months of storage at room temperature led to a higher germination index, while 29 months of storage at −18 °C increased the values compared with the 17 months of storage at room temperature where the germination index had low values even at the optimal temperature for milk thistle seeds germination (Table 4). Taking into account the interaction effect of the factors, the positive effect of cold storage on the germination index was more obvious in the population Mesopotamia reaching the values of the seeds that were stored only for 5 months, especially at 20 °C, 25 °C, and 30 °C.

### 2.4. Radicle and Hypocotyl Length

**Figure 4:**
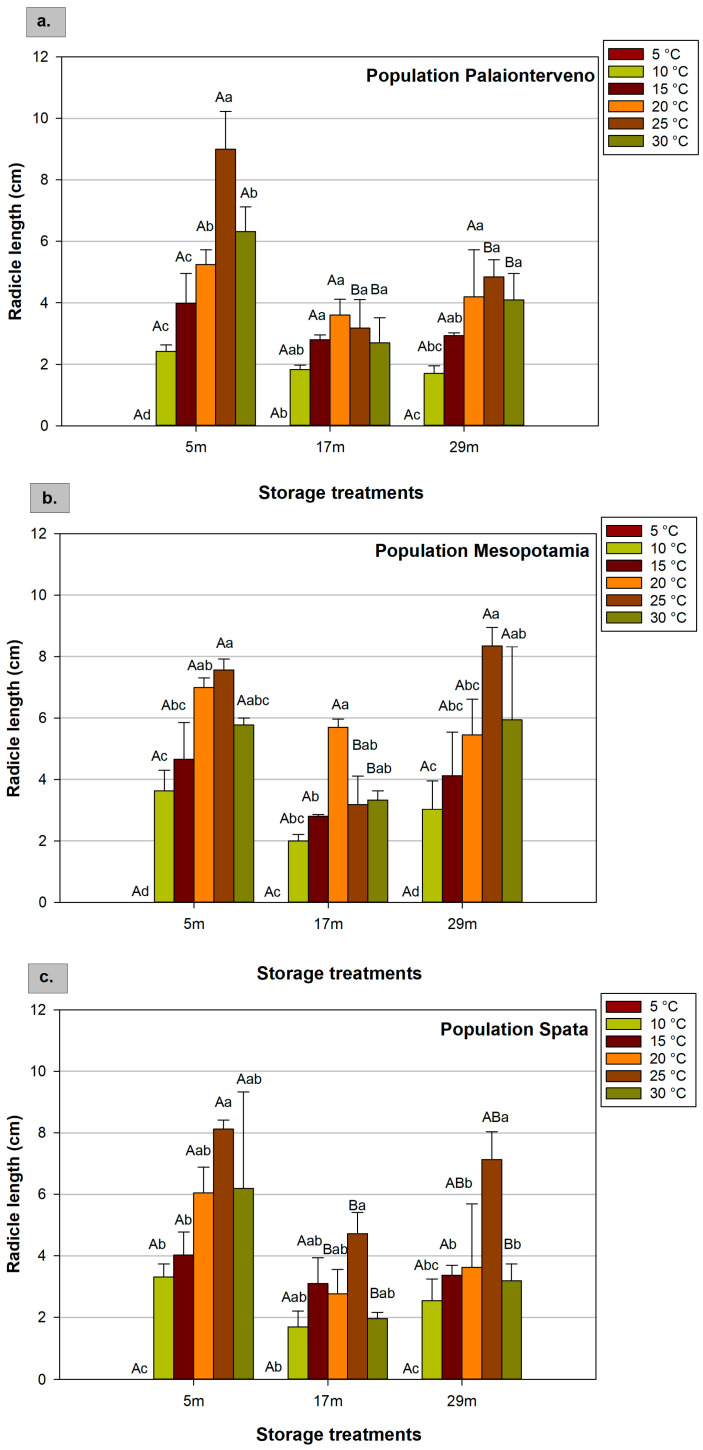
Effect of population ((**a**) Palaionterveno, (**b**) Mesopotamia and (**c**). Spata), storage (duration and conditions: 5, 17, and 29 refer to the months (m) of storage and the conditions that prevailed during storage), and temperature (5 °C, 10 °C, 15 °C, 20 °C, 25 °C, and 30 °C) on radicle length of milk thistle. Capital letters show significant differences between storage treatments at the same temperature, while small letters show significant differences between temperatures at the same storage treatment. The bars represent the standard error of the means.

**Figure 5:**
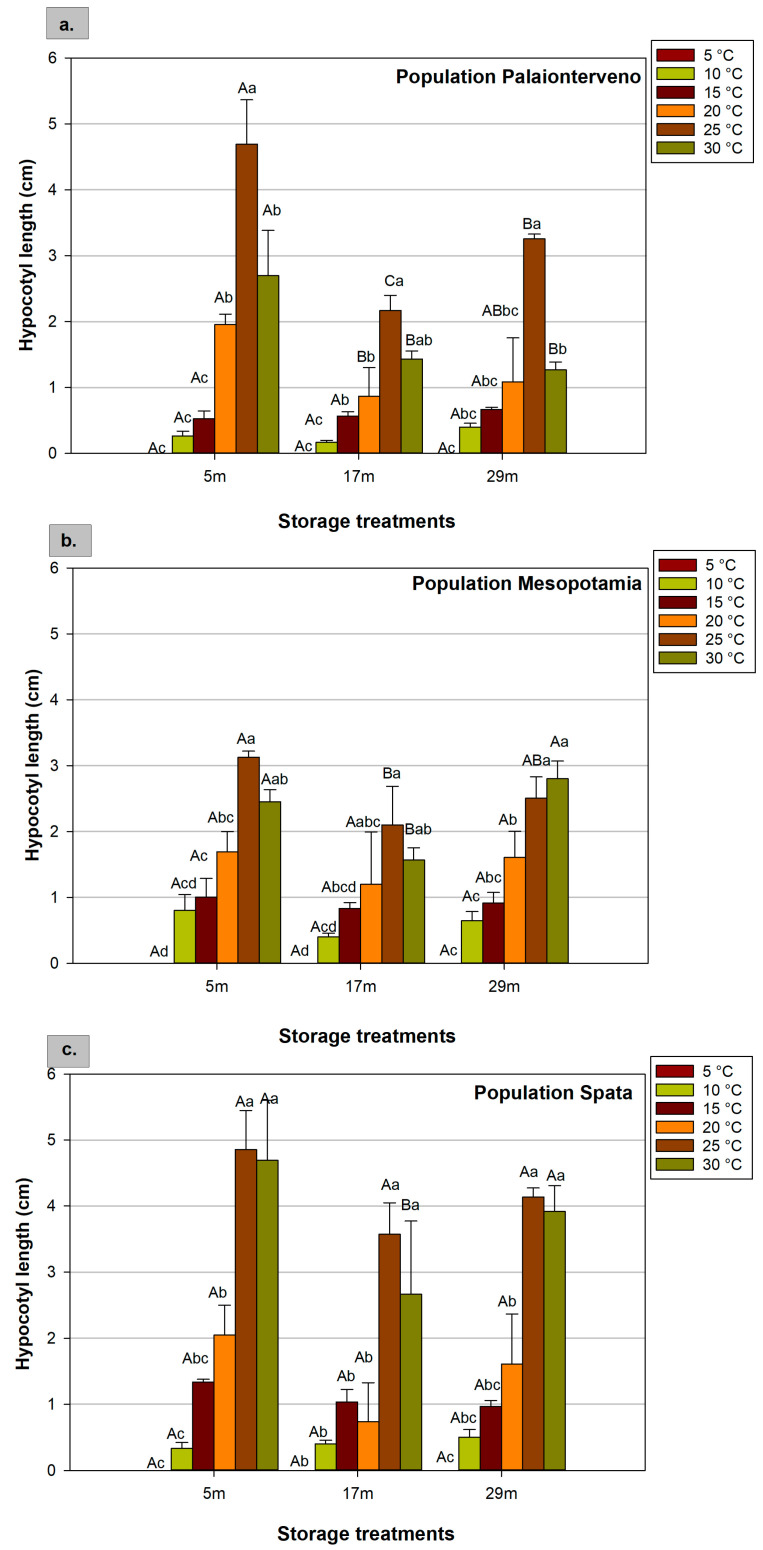
Effect of population ((**a**) Palaionterveno, (**b**) Mesopotamia, and (**c**) Spata), storage (duration and conditions: 5, 17, and 29 refer to the months (m) of storage and the conditions that prevailed during storage), and temperature (5 °C, 10 °C, 15 °C, 20 °C, 25 °C, and 30 °C) on hypocotyl length of milk thistle. Capital letters show significant differences between storage treatments at the same temperature, while small letters show significant differences between temperatures at the same storage treatment. The bars represent the standard error of the means.

The results showed that population, storage, and temperature affected the radicle and hypocotyl length (Figure 4 and Figure 5). As mentioned above, at 5 °C there was no seed germination and the measurement of radicle and hypocotyl length was not feasible. In terms of radicle length, in most cases, the maximum values were recorded at 25 °C, while at 10 °C and 15 °C, the radicle was smaller compared to higher temperatures. For instance, for seeds stored for 5 months, when evaluating the mean values of the three populations, the radicle length was 3.12 cm and 8.23 cm at 10 °C and 25 °C, respectively (Table 2), indicating the significant impact of temperature in this parameter. Assessing the storage conditions and duration, the radicle that developed from seeds stored for 5 months was bigger than the seeds stored for 17 months at room temperature. However, the cold storage at −18 °C for 29 months beneficially affected the radicle growth compared to the 17 month’ storage, especially in the population Mesopotamia at 25 °C and 30 °C. In general, the effect of the storage was more obvious at 25 °C and 30 °C (Figure 4), while at 20 °C no significant differences between the storage treatments in the mean values of the three populations (Table 4) and between the populations for the seeds that were stored for 5 months at room temperature, were found (Table 3).

Moreover, in the population Mesopotamia at 20 °C the development of radicle from seeds that were stored for 17 months was higher by 51% compared with the radicle of the population Spata at the same duration of storage (Figure 4b,c). In general, the highest length (8.99 cm) was observed in Palaionterveno seeds stored for 5 months at room temperature (Figure 4a).

Similarly with radicle length, the highest values of hypocotyl length were recorded at 25 °C, followed by 30 °C. At seeds that were stored for 5 months, assessing temperature as mean values of the three populations, hypocotyl length was 0.47 cm and 4.23 cm at 10 °C and 25 °C, respectively (Table 2). At 20 °C, there was no significant differences between the populations for the seeds that were stored for 5 months at room temperature (Table 3). However, regarding the interaction effect of three factors, at 25 °C and 30 °C, there were significant differences between Spata and the other populations in the hypocotyl length as the values were higher by up to 57%. In general, Spata had the maximum length (4.85 cm) with the exception of the 10 °C where the hypocotyl growth was limited (Figure 5c). At 20 °C, evaluating the mean values of the three populations, storage duration and conditions had no significant impact on hypocotyl length as the hypocotyl growth was not so strong at this temperature (Table 4). However, at higher temperatures (25 °C and 30 °C), prolonged storage seemed to inhibit hypocotyl growth and storage at −18 °C mitigated the reduction, especially at the optimum temperatures and in the Spata population. At 25 °C, the hypocotyl length that developed from the cold-stored seeds for 29 months increased by 33%, 16%, and 14% in the populations Palaionterveno, Mesopotamia, and Spata, respectively, compared with the hypocotyl that developed from the seeds that were stored for 17 months at room temperature (Figure 5).

## 3. Discussion

Seed germination is a characteristic highly depended by the genotype [16,26,27]. Our results reveal that milk thistle population affected the seed germination percentage. The highest percentage (96.7%) was recorded at 25 °C in the Mesopotamia population for the seeds that were stored for 5 months at room temperature. Differences in milk thistle seed germination percentage ascribed to population were also found in the study of Monemizadeh et al. [16]. However, in the present study a similar pattern between populations at the same temperature and storage condition was recorded. Moreover, storage duration and conditions had a significant impact on seed germination, while interactions between temperature and storage were noted. Seeds that were stored for 5 months at room temperature had a high germination percentage at 20 °C and 25 °C, while differences between the populations were found. Monemizadeh et al. [16] observed that 2-month after-ripened seeds had a higher germination percentage than 7-month after-ripened seeds, owing to seed aging or secondary dormancy. Storage of seeds for 17 months at room temperature negatively affected seed germination, while storage for 29 months at −18 °C slightly mitigated the adverse effect of long-term storage in Palaionterveno and Spata populations, while in Mesopotamia population led to high germination percentages. This can be ascribed to the delayed seed deterioration caused by storage under cooling conditions as found for soybean as well [26]. Seed aging and deterioration which are caused by various biochemical processes such as lipid peroxidation [32], DNA and RNA damage, and protein oxidation [33,34] delay seedling emergence [35], decline the germination ability [33], and reduce the seedling length [36]. Moreover, seed aging is affected by the storage conditions such as temperature and relative humidity [25,35]. Our results also show that storage of milk thistle seeds for 17 months at room temperature negatively affected radicle and hypocotyl length even when optimum temperatures for germination were applied.

In this study, temperature and storage significantly affected mean germination time, although the effect of temperature is more evident as the mean germination time is higher at lower temperatures. Similarly, Bhatt et al. [17] observed that temperature influences mean germination time and each plant species has specific requirements for germination speed. The mean germination time of milk thistle seeds ranged from 1.37 to 7.67 days and the lowest values were recorded at 25 °C. The mean germination time of other species was 3.3 to 4.3 days (at 25 °C) in einkorn [25] and 3.1 to 3.8 days in common bean (at 20 °C) [37]. Concerning the germination index, storage caused a significant reduction. At a temperature of 20 °C, the 17 months of storage at room temperature decreased germination index by up to 95% compared to the 5 month’ storage. Similarly, the germination index of African whitewood (*Triplochiton scleroxylon* K. Schum.) seeds decreased with storage, while a negative correlation between this index and storage period was recorded [38].

Our results revealed no germination at 5 °C regardless of the population and storage conditions applied. In a previous study, the base temperature for germination of milk thistle seeds was 5.2 °C [19], while Martin et al. [39] observed that milk thistle seedling’s emergence was less than 10% when the soil temperature at the depth of 10 cm was less than 13 °C. Base temperatures for seed germination vary between the weed species. For instance, the base temperatures of other broadleaf winter weeds are 4.7 °C for chamomile (*Matricaria chamomilla* L.), 2.5 °C for field poppy (*Papaver rhoeas* L.), 2.3 °C for chickweed (*Stellaria media* (L.) Vill.) [40], 4.5 °C for shepherd’s purse (*Capsella bursa-pastoris* (L.) Medik.), 0.2 °C for ivy-leaved speedwell (*Veronica hederifolia* L.) [41], and 2 °C for false cleavers (*Galium spurium* L.) [42]. The knowledge of the base temperatures for seed germination is crucial for weed management since they can be used in predicting weed emergence and planning the appropriate crop sowing date. In field experiments conducted in Greece, Karkanis et al. [3] observed that the late sowing of durum wheat after the middle of November significantly decreased the density of milk thistle compared to the early sowing, revealing the importance of sowing date as a sustainable integrated weed management method. The reduction in weed density in the study of Karkanis et al. [3] was ascribed to the lower temperatures recorded after the middle of November (late sowing) compared to those recorded in the first fortnight of November (early sowing).

The knowledge of weeds dynamics and seed losses is important when designing crop rotation schemes [43]. Recently, some studies evaluated the seed persistence of various weeds such as yellow foxtail (*Setaria pumila* (Poir.) Roem. & Schult.) [22], annual bastardcabbage (*Rapistrum rugosum* (L.) All.), common sowthistle (*Sonchus oleraceus* L.) [44], redroot pigweed (*Amaranthus retroflexus* L.), and slender amaranth (*Amaranthus viridis* L.) [23] indicating the growing interest in this area as the results about seed resistance of weeds can lead to optimization of control methods and integrated management programs [22,23,44]. To our knowledge, there are no studies about milk thistle seed persistence in the soil, although in this study, a reduction in germination rate was observed after 17 months of storage at room temperature. It is also important to mention that milk thistle seeds contain oil at a high percentage (22.1–25.1%) [4], a parameter that may be related to the decline in germination percentage over time. Usually, oily seeds exhibit shorter longevity [35], a trait that affects seed persistence [45]. Thus, the great decline in seed germination percentage after 17 months of storage indicates that the implementation of a three-year rotation system (spring crop–spring crop–winter crop) that includes crops with different life cycles can contribute to the reduction of milk thistle density in winter crops such as cereals and legumes. In another study, Koocheki et al. [43] reported that the rotation of winter wheat with spring crops such as maize or sugar beet led to a significant reduction of weed seed bank compared to that in the winter wheat monocropping system. It is also important to point out that by including milk thistle in a crop rotation system (e.g., milk thistle–maize–pea–durum wheat), Týr [46] observed that the seeds of milk thistle in the soil bank significantly reduced in pea and wheat crops that followed two and three years after the milk thistle crop, respectively.

Beyond weed management, seed persistence is also an important issue for crop establishment [45], since shorter longevity and low germination percentage of seeds lead to crop yield decline due to lower plant density as a result of poor seedling emergence. Information about seed germination of milk thistle is important for crop establishment since this species is also cultivated as a medicinal plant. Our results show that regardless of the populations and storage conditions, crop establishment is not feasible at low temperatures (5 °C) since under these conditions no germination of seeds was observed. Moreover, seeds that were stored for 5 months at room temperature had efficient germination percentages at a wide range of temperatures, from 15 °C to 30 °C, especially in the population Mesopotamia followed by the population Spata, while the population Palaionterveno had lower percentages. In terms of storage, seeds that were stored for 17 months at room temperature had low germination percentages. However, storage for 29 months at −18 °C positively affected the germination compared with 17 months of storage. Kurek et al. [33] mentioned that seed aging and the loss of viability could lead to a lack of reproduction material. Thus, the knowledge of the optimum storage conditions of milk thistle seeds is a crucial factor to delay seed aging and the adverse effects of this process on crop establishment.

## 4. Materials and Methods

### 4.1. Seed Sampling, Storage, Germination Test, and Temperature Treatments

Three milk thistle wild populations originating from Greece (Factor A) were used in this study: Palaionterveno, Mesopotamia, and Spata. The selection of the populations was based on silymarin content as determined in a previous study by Arampatzis et al. [47]. The Population Spata has high silymarin content, silybin A, and silybin B, while the populations Palaionterveno and Mesopotamia have lower silymarin content but similar silymarin composition [47]. These populations were cultivated under the same environmental conditions at the experimental farm of University of Thessaly in Velestino (Longitude: 22.7565, Latitude: 39.3963). Sowing took place at the end of October (2017, 2018, and 2019) in rows 50 cm apart (plant density: 26 plants m^−2^). Four rows, 3 m long, were sown for each population, while weed control was carried out by hand hoeing on 14 February 2018, 19 February 2019, and 18 February 2020. The meteorological data in the Velestino region during the growth period (November to May) of milk thistle are previously presented by Arampatzis et al. [48] and Karkanis et al. [3]. The three populations were hand harvested at the same period. The central capitulum from 100 plants of each population was randomly collected on 27 May to 3 June 2018, 26 to 30 May 2019, and 25 to 30 May 2020 when the plants reached the ripening growth stage 89 (BBCH scale) as described by Martinelli et al. [49]. After the harvest, the seeds were separated from the capitulum and stored for different durations and under different storage conditions (Factor B). In particular, seeds collected in 2020, 2019, and 2018 were stored for 5 months at room temperature, 17 months at room temperature, and 29 months in the freezer at −18 °C, respectively.

**Figure 6:**
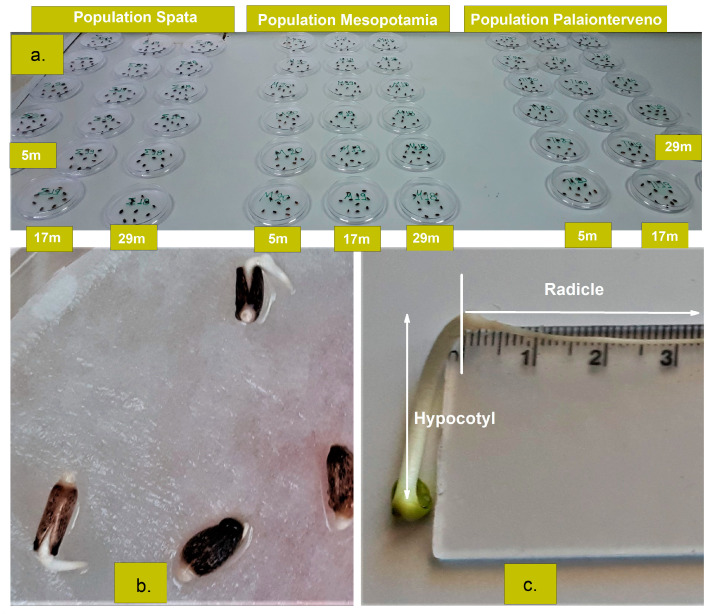
(**a**). Arrangement of the Petri dishes (diameter 9 cm, 10 seeds/dish, 60 seeds/treatment) for each milk thistle population (Spata, Mesopotamia, and Palaionterveno) before placement in the germination chamber (5, 17, and 29 refer to the months (m) of storage and the conditions that prevailed during storage). The equal number (54) of Petri dishes was used at each of the six tested temperatures (5 °C, 10 °C, 15 °C, 20 °C, 25 °C, and 30 °C). (**b**) Germinated seeds of milk thistle, and (**c**) Hypocotyl and radicle of seedling.

The germination test was performed on sterile Petri dishes (diameter 9 cm, 10 seeds/dish) according to a completely randomized design with three replications for each experimental treatment (60 seeds/treatment, Figure 6a). Before the germination test, seeds were surface-sterilized in a solution of 5% sodium hypochlorite for 5 min and then washed five times with distilled water. The sterilized seeds were placed in the Petri dishes containing Whatman No.1 filter paper disks and soaked with distilled water. At regular intervals during the germination test, distilled water was added to keep the filter paper at the base of the Petri dishes and to keep the seeds moist [50]. To assess the effect of temperature on milk thistle germination (Factor C), the incubation took place at six different temperatures (5 °C, 10 °C, 15 °C, 20 °C, 25 °C, and 30 °C) in a seed germination chamber (Raypa, Model IRE-160, Terrassa (Barcelona), Spain) under dark conditions.

### 4.2. Measurements

#### 4.2.1. Germination Percentage

Seeds with a radicle length of 2 mm (Figure 6b) were considered germinated [51]. These seeds were counted at the same time every day and the germination percentage (GP) was calculated by Equation (1) [52]:

(1) $$\mathrm{GP}\;=\frac{\mathrm{Number}\;\mathrm{of}\;\mathrm{germinated}\;\mathrm{seeds}}{\mathrm{Total}\;\mathrm{number}\;\mathrm{of}\;\mathrm{seeds}}\times 100$$

#### 4.2.2. Mean Germination Time 

Mean germination time (MGT) was calculated using Equation (2) as suggested by Ellis and Roberts [53]:

(2) $$\mathrm{MGT}=\frac{\sum^{\;}\left(\mathrm{ni}\times \;\mathrm{di}\right)}{N}$$

where ni is the number of seeds germinated at day i from the beginning of the germination test and N is the total number of germinated seeds at the end of the test.

#### 4.2.3. Germination Index

Germination Index (GI) was estimated according to Equation (3) [54]:

(3) $$\mathrm{GI}=[\frac{\mathrm{Number}\;\mathrm{of}\;\mathrm{germinated}\;\mathrm{seeds}\;\mathrm{in}\;\mathrm{first}\;\mathrm{count}}{\mathrm{Days}\;\mathrm{of}\;\mathrm{fisrt}\;\mathrm{count}}]\;+\;\ldots \;+\;[\frac{\mathrm{Number}\;\mathrm{of}\;\mathrm{germinated}\;\mathrm{seeds}\;\mathrm{in}\;\mathrm{final}\;\mathrm{count}}{\mathrm{Days}\;\mathrm{of}\;\mathrm{final}\;\mathrm{count}}]$$

#### 4.2.4. Radicle and Hypocotyl Length

Radicle and hypocotyl length (Figure 6c) were measured 7 days after the initiation of germination in all the germinated seeds of each Petri dish.

### 4.3. Statistical Analysis

The experimental data were analyzed using the SigmaPlot 12 statistical package (Systat Software, San Jose, CA, USA). A three-way analysis of variance (ANOVA) was conducted to evaluate the effects of three factors (population, duration-storage conditions, and temperature) and their interactions on seed germination parameters. Duncan’s multiple-range test at a *p* ≤ 0.05 was used to assess the significance of the differences between means. Data are presented in figures as means ± SE (Standard error) of three replications.

## 5. Conclusions

The results of this study indicate that milk thistle population, duration and storage conditions, and temperature significantly affected seed germination. The highest germination percentages and germination indexes were recorded for seeds that were stored for 5 months at room temperature, while long-term storage of milk thistle seeds led to reduced values. However, 29 months of storage at −18 °C increased the values compared with 17 months at room temperature revealing that cold storage could mitigate the adverse effects. In the population Mesopotamia the influence of cold storage was more evident than in other populations. The highest germination was observed at 20 °C and 25 °C and particularly at 25 °C in the Mesopotamia population for the seeds that were stored for 5 months at room temperature. Milk thistle can germinate to a wide range of temperatures, but not at 5 °C. In general, lower temperatures increased mean germination time and decreased radicle and hypocotyl length, while the effects of population and storage are apparent. At optimum temperature and after 5 months of storage, the germination percentage of the three populations ranged from 73.3% to 96.7% and the mean germination time was 1.37 to 3.08 days. The different behavior of populations at storage and temperature regimes indicate the importance of selecting the appropriate genetic material, the storage conditions, and the sowing date to achieve an efficient crop establishment. Moreover, the knowledge of milk thistle germination and the loss of viability through time is crucial to develop integrated weed management programs.

## Figures and Tables

**Table 1:** Three-way ANOVA results of germination percentage (GP), mean germination time (MGT), germination index (GI), radicle length (RL), and hypocotyl length (HL).

Factors	F Values of ANOVA				
	GP	MGT	GI	RL	HL
Population (P)	27.848 ***	7.007 ***	30.562 ***	3.808 *	13.826 ***
Storage (S)	220.704 ***	38.445 ***	273.201 ***	25.902 ***	16.958 ***
Temperature (T)	90.281 ***	351.308 ***	128.943 ***	54.827 ***	113.374 ***
P × S	12.784 ***	2.504 *	10.566 ***	0.987 ^ns^	0.768 ^ns^
P × T	3.728 ***	2.366 *	5.054 ***	0.831 ^ns^	5.300 ***
S × T	14.236 ***	2.964 **	30.257 ***	2.873 **	2.782 **
P × S × T	2.937 ***	1.306 ^ns^	3.810 ***	0.619 ^ns^	0.696 ^ns^

*, **, and *** significant at *p* ≤ 0.05, *p* ≤ 0.01, and *p* ≤ 0.001, respectively. ns = not significant.

**Table 2:** Effects of temperature (5 °C, 10 °C, 15 °C, 20 °C, 25 °C, and 30 °C) on milk thistle seeds’ germination percentage (GP), mean germination time (MGT), germination index (GI), radicle length (RL), and hypocotyl length (HL). Seeds were stored for 5 months at room temperature (mean values of three populations).

Temperature	GP (%)	MGT (Days)	GI	RL (cm)	HL (cm)
5 °C	0 d	0 e	0 e	0 d	0 e
10 °C	41.1 c	5.61 a	5.53 d	3.12 c	0.47 de
15 °C	60.0 b	3.33 b	13.49 c	4.22 bc	0.96 d
20 °C	75.6 a	2.69 bc	19.74 b	6.09 ab	1.90 c
25 °C	83.9 a	1.73 d	32.07 a	8.23 a	4.23 a
30 °C	48.9 bc	2.12 cd	15.00 bc	6.10 ab	3.28 b

For each column, mean values followed by same letters show no significant differences between the treatments.

**Table 3:** Effects of milk thistle populations (Palaionterveno, Mesopotamia, and Spata) on seeds’ germination percentage (GP), mean germination time (MGT), germination index (GI), radicle length (RL), and hypocotyl length (HL) at 20 °C. Seeds were stored for 5 months at room temperature.

Populations	GP (%)	MGT (Days)	GI	RL (cm)	HL (cm)
Palaionterveno	73.3 a	2.51 a	19.70 a	5.24 a	1.96 a
Mesopotamia	76.7 a	3.02 a	18.66 a	6.99 a	1.69 a
Spata	76.7 a	2.84 a	20.87 a	6.05 a	2.05 a

For each column, mean values followed by same letters show no significant differences between the treatments.

**Table 4:** Effects of storage duration and conditions (5, 17, and 29 refer to the months (m) of storage and the conditions that prevailed during storage) on milk thistle seeds’ germination percentage (GP), mean germination time (MGT), germination index (GI), radicle length (RL), and hypocotyl length (HL) at 20 °C (mean values of three populations).

Storage Treatments	GP (%)	MGT (Days)	GI	RL (cm)	HL (cm)
5 m	75.6 a	2.69 a	19.74 a	6.09 a	1.90 a
17 m	15.0 c	3.98 a	2.39 c	4.02 a	0.93 a
29 m	43.3 b	3.38 a	10.47 b	4.42 a	1.43 a

For each column, mean values followed by same letters show no significant differences between the treatments.

## Data Availability

The data presented in this study are available in this article.
